# Modulating the Gut Microbiota Improves Glucose Tolerance, Lipoprotein Profile and Atherosclerotic Plaque Development in ApoE-Deficient Mice

**DOI:** 10.1371/journal.pone.0146439

**Published:** 2016-01-22

**Authors:** Ida Rune, Bidda Rolin, Christian Larsen, Dennis Sandris Nielsen, Jenny E. Kanter, Karin E. Bornfeldt, Jens Lykkesfeldt, Karsten Buschard, Rikke Kaae Kirk, Berit Christoffersen, Johannes Josef Fels, Knud Josefsen, Pernille Kihl, Axel Kornerup Hansen

**Affiliations:** 1 Section of Experimental Animal Models, Department of Veterinary Disease Biology, Faculty of Health and Medical Sciences, University of Copenhagen, Thorvaldsensvej 57, 1871 Frederiksberg C, Denmark; 2 Global Research, Novo Nordisk A/S, Novo Nordisk Park 1, 2760 Måløv, Denmark; 3 Department of Food Science, Faculty of Science, University of Copenhagen, Rolighedsvej 26, 1958 Frederiksberg C, Denmark; 4 Department of Medicine, Diabetes and Obesity Center of Excellence, University of Washington, Seattle, WA, 98109, United States of America; 5 The Bartholin Institute, Rigshospitalet Dept. 3733, Copenhagen Biocenter, Ole Maaløes Vej 5, 2200 København N, Denmark; University of Catanzaro Magna Graecia, ITALY

## Abstract

The importance of the gut microbiota (GM) in disease development has recently received increased attention, and numerous approaches have been made to better understand this important interplay. For example, metabolites derived from the GM have been shown to promote atherosclerosis, the underlying cause of cardiovascular disease (CVD), and to increase CVD risk factors. Popular interest in the role of the intestine in a variety of disease states has now resulted in a significant proportion of individuals without coeliac disease switching to gluten-free diets. The effect of gluten-free diets on atherosclerosis and cardiovascular risk factors is largely unknown. We therefore investigated the effect of a gluten-free high-fat cholesterol-rich diet, as compared to the same diet in which the gluten peptide gliadin had been added back, on atherosclerosis and several cardiovascular risk factors in apolipoprotein E-deficient (*Apoe*^*-/-*^) mice. The gluten-free diet transiently altered GM composition in these mice, as compared to the gliadin-supplemented diet, but did not alter body weights, glucose tolerance, insulin levels, plasma lipids, or atherosclerosis. In parallel, other *Apoe*^*-/-*^ mice fed the same diets were treated with ampicillin, a broad-spectrum antibiotic known to affect GM composition. Ampicillin-treatment had a marked and sustained effect on GM composition, as expected. Furthermore, although ampicillin-treated mice were slightly heavier than controls, ampicillin-treatment transiently improved glucose tolerance both in the absence or presence of gliadin, reduced plasma LDL and VLDL cholesterol levels, and reduced aortic atherosclerotic lesion area. These results demonstrate that a gluten-free diet does not seem to have beneficial effects on atherosclerosis or several CVD risk factors in this mouse model, but that sustained alteration of GM composition with a broad-spectrum antibiotic has beneficial effects on CVD risk factors and atherosclerosis. These findings support the concept that altering the microbiota might provide novel treatment strategies for CVD.

## Introduction

GM composition has been shown to be altered and likely impact the development and progression of numerous conditions including atherosclerosis, obesity, diabetes, and, maybe less surprisingly, coeliac disease (gluten intolerance) [1–5].One of the links connecting GM to systemic inflammation, and thereby indirectly to development of atherosclerosis, is activation of the Toll-like receptors (TLR) in the intestine resulting in increased secretion of pro-inflammatory cytokines [6]. TLR-4 is mainly stimulated by lipopolysaccharide (LPS) originating from Gram-negative bacteria, but other GM-derived particles are also capable of stimulating members of the TLR-family [6,7] and promote atherosclerosis in mouse models [5,8]. High fat diets have been shown to favour colonization of Gram-negative microbiota in the gut leading to increased production of LPS [9]. High levels of circulating LPS are correlated to increased macrophage infiltration in adipose tissue and systemic inflammation, adding to the risk of developing for instance atherosclerosis [10]. Karlsson et al. suggest a more direct association between the gut microbiota and the inflammatory state of atherosclerotic patients, where bacteria from the intestinal lumen is phagocytosed by macrophages and then transported directly to sites of endothelial inflammation, where they along with the macrophage is incorporated into the atherosclerotic lesions [11]. Thus, in addition to well-known cardiovascular risk factors, such as dyslipidemia, hypertension and insulin resistance, the GM composition has been suggested to alter CVD risk in human subjects.

The realization that alterations of GM composition and intestinal changes are possible contributors to a number of diseases has attracted significant interest, not only in the scientific community but also in the society as a whole. This notion has contributed to the large increase in individuals who have switched to gluten-free diets in the belief that gluten is responsible for many of the common chronic diseases of our time[12]. Whereas it is clear that a gluten-free diet benefits patients with coeliac disease and possibly the pre-diabetic state of type 1 diabetes (T1D) [13], the effects of a gluten-free diet on other types of diseases, such as atherosclerosis, is largely unknown. In both mice [14] and humans [15] it has been shown that dietary gluten reduces the abundance of bacteria capable of stimulating the immune system, which in animal models have been shown to increase the risk of development of inflammatory disease, such as type 1 diabetes (T1D) [16]. In the gut, both dietary gluten and GM-derived particles have been suggested to interact with TLRs causing a pro-inflammatory innate immune response [6,7,17–19]. In comparison with other peptides, the gluten peptide, gliadin, is more resistant to enzymatic degradation, which has been suggested to result in prolonged immune-stimulation, leading to more inflammation [19,20].

In this study, we investigated the effect of gluten-free diet, as compared to the same diet supplemented by the gluten protein, gliadin, on clustering of GM, several cardiovascular risk factors and atherosclerosis in *Apoe*^*-/-*^ mice. The broad-spectrum antibiotic ampicillin, which markedly alters GM, was used as a positive control. Our results demonstrate that a gluten-free diet does not seem to have beneficial effects on atherosclerosis or several CVD risk factors, but that sustained alteration of GM composition with ampicillin has beneficial effects on CVD risk factors and atherosclerosis.

## Materials and Methods

### Ethics statement

Experiments were performed in accordance with the EU directive 2010/63/EU on the Protection of Vertebrate Animals used for Scientific Purposes, and the Danish Animal Experimentation Act (LBK 1306 from 23/11/2007 with 2011 amendments). The study was approved by the Animal Experiments Inspectorate, Ministry of Food and Environment, Denmark.

### Animals

Because microbiota is transferred from female mice to her pups, the different diets were administered to pregnant mice in order to obtain more reliable results from their pups. Twenty pregnant B6.129P2-*Apoe*^*tm1Unc*^ N11 (*Apoe*^*-/-*^) mice (Taconic Europe A/S, Ejby, Denmark) were randomly divided into four different treatment groups each consisting of five animals. Each group was fed one of the four diets described below. The single-housed pregnant mice gave birth to 45 female pups, which at four weeks of age were randomized into cages containing four to six animals within their respective group. Animals were kept at room temperature in a twelve hour light cycle. The study continued for a total of 16 weeks from the birth of the pups. Animals were weighed weekly from weaning at week four. Prior to euthanasia by saline perfusion through the left cardiac ventricle, animals were anaesthetized with a Hypnorm/Dormicum mixture (0.3 ml SC in a 1:1:2 water solution, Vetapharm Ltd., Sherburn in Elmet, Leeds, UK; Roche A/S, Hvidovre, Denmark). Animals were subjected to daily visual control and animals showing signs of illness or misthriving were monitored by an affiliated veterinarian.

### Diets and antibiotic treatment

Animals in group one received Western Diet without gluten (D12079B, Research Diets Inc., New Brunswick, NJ, USA) and pure tap water. Animals in group two received the same gluten-free Western Diet supplemented with gliadin (Cat. #D11061501, Research Diets Inc., New Brunswick, NJ, USA with 1% gliadin; Cat. #G3375, Sigma, St. Louis, MO, USA) and pure tap water (Table 1). Animals in group three received gluten-free Western Diet containing 1% gliadin and tap water containing the broad spectrum antibiotic ampicillin (1 g/L) (Ampivet^®^ vet., Boehringer Ingelheim, Copenhagen, Denmark). Animals in group four received gluten-free Western Diet and tap water containing ampicillin. The dietary concentration of the gluten-peptide, gliadin, was chosen to best mimic that of ordinary barley-based rodent chow [21]. The diets were weighed and changed twice weekly. Water was changed twice weekly. Diets and treatment were consistent throughout the study.

**Table 1:** Composition of groups.

		Ampicillin	
		-	+
**Dietary Gliadin**	**-**	WD (n = 12)	WD+A (n = 12)
	**+**	WD+G (n = 9)	WD+A+G (n = 12)

Group 1 (WD); Animals received gluten-free Western Diet and tap water. Group 2 (WD+G); Animals received gluten-free Western Diet with added gliadin and tap water. Group 3 (WD+G+A); Animals received gluten-free Western Diet with added gliadin and ampicillin in their drinking water. Group 4 (WD+A); Animals received gluten-free Western Diet and ampicillin in their drinking water.

### Gut microbiota composition

Fecal samples obtained aseptically at week 5 and 16 were analysed by denaturing gradient gel electrophoresis (DGGE) as previously described by Hufeldt, with a few alterations [22]. In brief, bacterial DNA was extracted using the QIAamp DNA Stool Mini Kit (Qiagen, Hilden, Germany). Prior to extraction, samples were homogenized using a FastPrep^®^-24 (MP, Biomedicals, Kem-En Tec A/S, Taastrup, Denmark) for 3 x 20 sec at 5.5 m/sec. Quality and concentration of the extracted DNA was verified on a NanoDrop 1000 Spectrophotometer (Thermo Scientific, USA). Genetic material was amplified by Polymerase Chain Reaction (PCR), using primers specific to the V3 region of the 16S rRNA gene. Genetic material was then separated by means of DGGE on a polyacrylamide gel containing a 30–65% chemical gradient (100% corresponds to 7 M urea and 40% formamide). DGGE profiles were analysed using BioNumerics Version 7.1 (Applied Maths, Sint-Martens-Latem, Belgium) for cluster analysis [Dice similarity coefficient with a band position tolerance and optimization of 1% using the Unweighted Pair Group Method with Arithmetic averages clustering algorithm (UPGMA) and Principal Component Analysis (PCA)]. GM composition results are presented as PCA-plots obtained from generated dendrograms.

### Glucose and Insulin

Glucose tolerance was evaluated by means of oral glucose tolerance test (OGTT) three times during the study; at 5, 11 and 16 weeks of age. Mice were fasted for four hours prior to testing and given a glucose solution (2 g/kg, Fresenius Kabi, Copenhagen, Denmark, concentration 500 g/l) by oral gavage. Blood samples were collected at *t* = 0, 30, 60, 90, 120 and 180 minutes post gavage. Glucose levels were measured by transferring 5 μl whole-blood to 500 μl EBIO buffer and analysing glucose levels by a Biosen glucose auto analyser (Eppendorf, Hamburg, Germany).

Insulin levels were measured just prior to glucose dosing at all three time points and again at 30 minutes post dosing in the 16 weeks old animals. Plasma samples were analysed for insulin using the "Ultra-sensitive rat insulin ELISA kit" (Crystal Chem, Downers Grove, IL, USA) with the modifications that in-house rat insulin standards, prepared using heat-treated rat plasma, were used.

### Total plasma Cholesterol (TPC) and lipoprotein fractions

TPC and lipoprotein fractions were analysed at the Department of Pathology/Lipid Sciences, Wake Forrest University School of Medicine, Winston-Salem, NC, USA. To obtain sufficient volumes samples were pooled; two animals per pool and four pools corresponding to a total of eight animals per group were measured. TPC levels were measured using colorimetric enzymatic assays as previously described [23–25]. An aliquot of plasma containing approximately 20 μg of TPC was diluted in phosphate buffered saline (PBS) into a final volume of 400 μL for lipoprotein measurement. Samples were injected onto a Superose 6 HR 10/30 (Amersham Pharmacia) chromatography column after centrifugation and were run at 0.4 mL/min. The signal was integrated using Chrom Perfect Spirit Software (Justice Laboratory Software). By multiplying the TPC concentration by the cholesterol percentage within the elution region for each lipoprotein class VLDL-, LDL-, and HDL-cholesterol were determined.

### Biomarker of oxidative stress

Malondialdehyde (MDA) was used as a biomarker of lipid oxidation and was measured in plasma by HPLC with fluorescent detection as previously described [26].

### En face aortic plaque assessment

In connection with euthanasia, heart and aorta were gently flushed with 10 ml 0.9% saline. The thoracic aorta from the base of the heart to the 7^th^ rib was mechanically cleaned of visceral fat under light microscope, cut open, and placed on glass slides. Slides were photographed under a microscope and images were subsequently analysed using an image analysis tool (Visiomorph^™^, VIS—Visiopharm Integrator System, Hoersholm, Denmark). Plaque burden was expressed percentagewise as plaque area divided by the total tissue area.

### Histological analysis of neutral lipid accumulation in the aortic sinus

After trans-cardiac perfusion, the heart and 1–2 mm of the aortic root were fixed in 10% buffered formalin for 24 hours and then transferred to 20% sucrose in PBS for 24 hours. The hearts were trimmed by removing the lower part of the heart with the cutting axis perpendicular to the aortic root. The trimmed hearts were placed in a cryo mold with the cut surface at the bottom of the mold, cryo fixed in OCT compound (Tissue-Tek, Sakura Finetek, Copenhagen, Denmark) and frozen on dry ice. Sections were cut and discarded until the appearance of the aortic valve. Hereafter, all sections were cut at 10 μm thickness and were collected and placed on numbered glass slides (SuperFrost^®^Plus, Hounisen, Skanderborg, Denmark), three on each glass slide with a distance of 150 μm between the sections, a total of 15 slides covering a distance of 450 μm. Three slides with three sections on each slide from every animal were selected for oil red O staining (Sigma-Aldrich). The first slide was selected at random between slide no. 1 to 5 and the next two slides were selected 5 and 10 slides apart. The selected oil red O-stained glass slides were scanned on a digital slide scanner (Hamamatsu NanoZoomer 2.0 HT) at a 40X magnification and the oil red O-stained area was measured using an image analysis tool (Visiomorph^™^). The percent of oil red O-stained area was calculated as mean of the stained area divided by the total aortic cross-sectional area of the 9 sections.

### Statistics

All statistics were calculated using GraphPad Prism 6 (GraphPad Software, San Diego, CA, USA). Gaussian distribution of the data was measured with D’Agostino & Pearson omnibus normality test. Data assuming Gaussian distribution were analysed by two-way analysis of variance (ANOVA) with source of variation. Data not assuming Gaussian distribution were analysed by Kruskal-Wallis test with Dunn’s multiple comparisons test using WD as control group. A p-value < 0.05 was considered significant. Statistical analysis of body weight and OGTT was performed on area under the curve (AUC) values. AUC data over time was analysed with paired t-test when assuming Gaussian distribution and with Wilcoxon matched-pairs signed rank test when failing to assume Gaussian distribution. Statistical analysis of GM composition was performed on entry coordinates obtained from PCA. Samples were compared in a multidimensional manner, and PCA plots depict samples using the three axes with biggest differences in the entry-coordinates of the single sample. These entry coordinates were used for statistical analysis, and groups were considered different if they were significantly different in minimum one of the three axes (X, Y, Z equalling P1, P2 and P3). Insulin values and plasma cytokine values below detectable limits were given the value of half the lower limit of quantification (LLOQ).

## Results

### Ampicillin was a strong modulator of GM composition in the presence and absence of the gluten protein gliadin, whereas a gluten-free diet lost its impact on GM over time in *Apoe*^*-/-*^ mice

To avoid biased clustering of the GM samples simply due to animals being housed together, animals within each group were divided into more than one cage. In addition, fecal samples were randomized prior to being analysed to avoid biased clustering depending on which gel the samples were run on.

**Fig 1:**
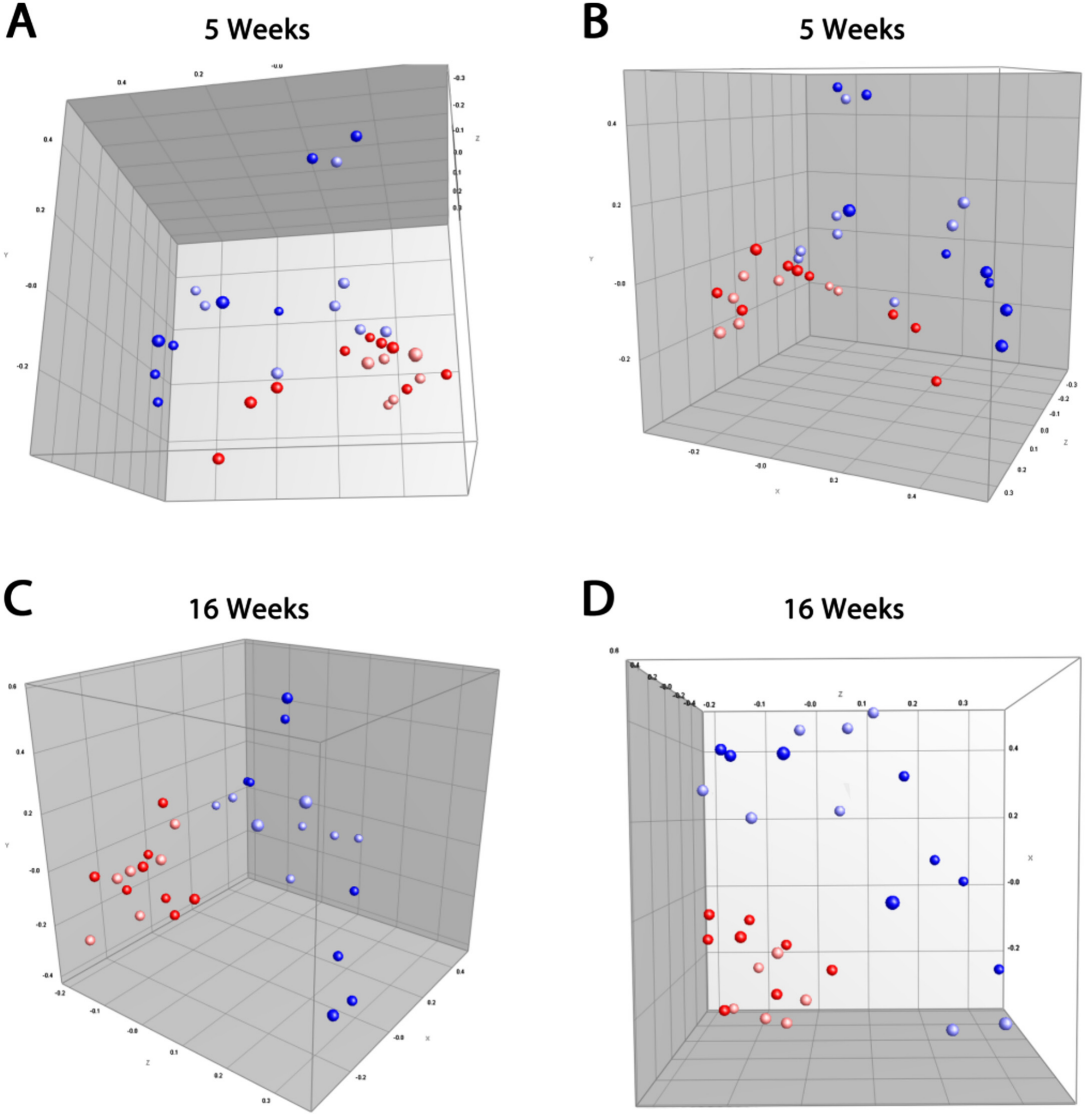
A gluten-free diet results in transient effects on the clustering of gut microbiota, whereas ampicillin has sustained effects. **A-B** Principal component analysis (PCA) plots of the GM at 5 weeks of age showing significantly differences between the groups depending on both gliadin and ampicillin-treatment (PC1; Gliadin: *p* = 0.0197 and ampicillin: *p* = 0.0005, PC2; Ampicillin: *p* = 0.0012). **C-D** PCA plots of the GM at 16 weeks of age showing significantly differences between the groups depending on ampicillin treatment (PC1; Ampicillin: *p* = 0.0033, PC3; Ampicillin: *p* = 0.0355). (Red: WD, light red: WD+G, light blue: WD+G+A, blue: WD+A).

When analysing entry coordinates obtained from the PCA plots, both ampicillin-treatment (PC1: *p* = 0.0005 and PC2: *p* = 0.0012) and dietary gliadin (PC1: *p* = 0.0197) strongly influenced GM in the young animals at five weeks of age (Fig 1A and 1B). When the mice reached 16 weeks of age, gliadin had lost its impact on GM composition, and only clustering depending on ampicillin-treatment was significant (PC1: *p* = 0.0033 and PC3: *p* = 0.0355, Fig 1C and 1D). These results showed that the broad-spectrum antibiotic ampicillin had marked and sustained effects on the GM composition, whereas a gluten-free diet had only transient effects.

### Ampicillin-treated *Apoe*^*-/-*^ mice gained more weight than untreated controls, whereas a gluten-free diet had no effect

**Fig 2:**
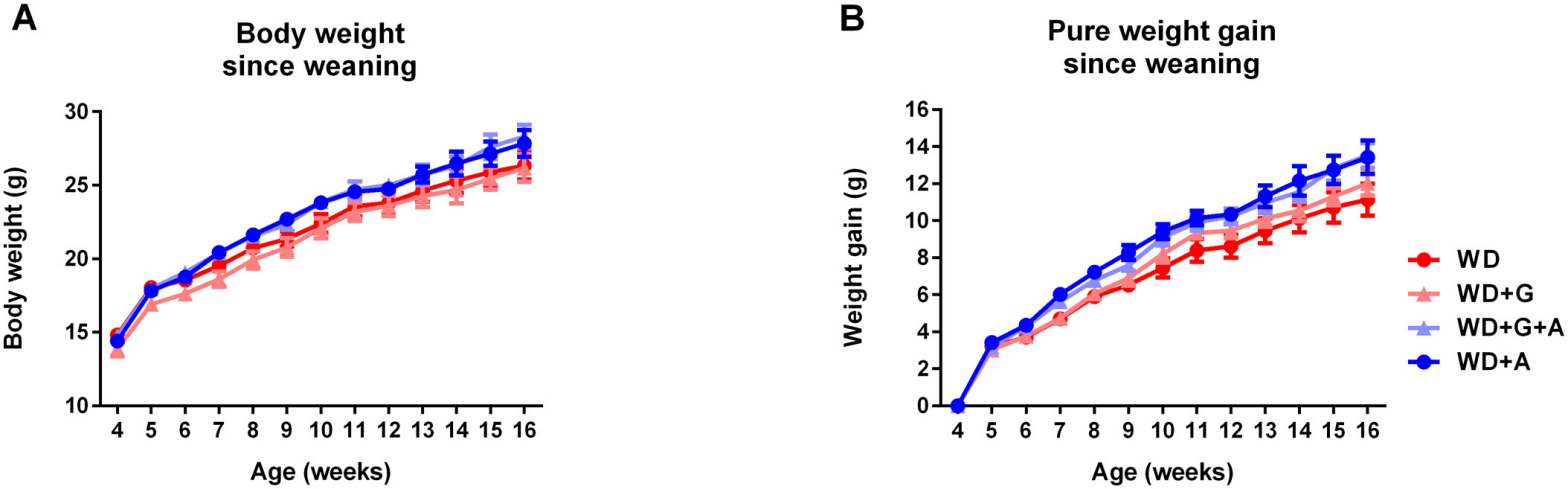
Body weights are increased by ampicillin, but not by a gluten-free diet. **A** Total body weight (g) from weaning until study termination at 16 weeks of age. **B** Pure weight gain from 5 weeks of age until termination at 16 weeks of age. Animals receiving ampicillin in their drinking water (WD+G+A and WD+A) had significantly higher body weight and pure weight gain (both AUC) compared to non-treated animals (*p* = 0.0366 and *p* = 0.0421, respectively). (Graphs depicted with mean ± SEM).

Ampicillin treated animals had slightly but significantly higher body weights as well as weight gain than untreated animals (*p* = 0.0366 and *p* = 0.0421 respectively, Fig 2A and 2B). The gluten-free diet had no effect on body weight gain.

### Ampicillin-treatment resulted in a transient improvement of glucose tolerance and reduced insulin levels in *Apoe*^*-/-*^ mice, whereas a gluten-free diet had no effect

**Fig 3:**
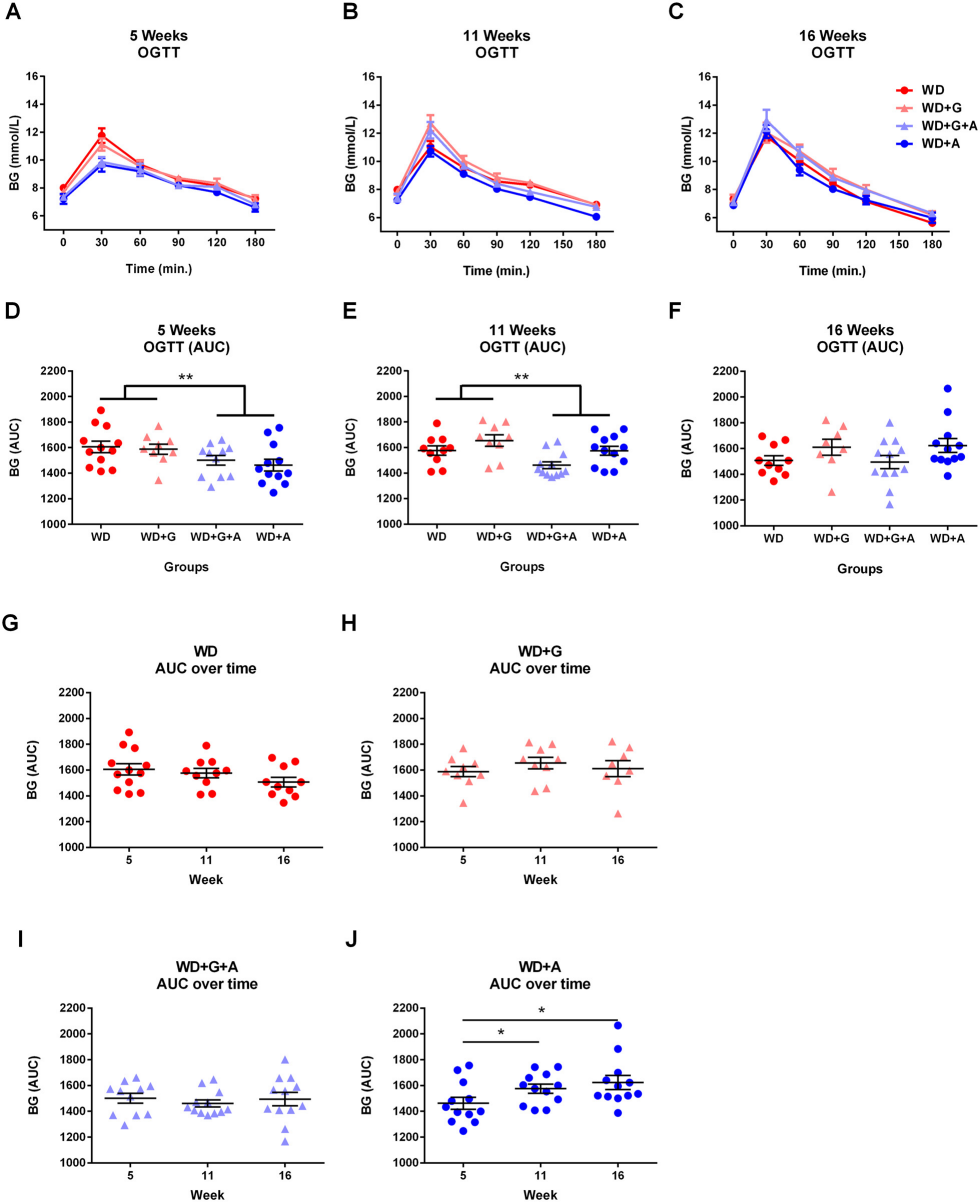
Glucose tolerance is transiently improved by ampicillin, but not by a gluten-free diet. **A-C** Glucose levels during oral glucose tolerance tests. **D-F** Oral glucose tolerance test performed at 5 and 11 weeks of age showed significant difference between the groups with ampicillin-treated animals having lower AUC levels than untreated animals (*p* = 0.0114 and *p* = 0.0095 respectively), whereas no differences could be demonstrated between the groups at 16 weeks of age. Gliadin did not influence glucose tolerance at any time point. **G-J** Development of glucose tolerance (AUC data) in the four groups over time show no difference over time in the WD, WD+G and WD+G+A groups but significant better glucose tolerance in the young ampicillin-treated animals (WD+A) at 5 weeks of age compared to when they are 11(*p* = 0.0210, Wilcoxon matched-pairs signed rank test) and 16 (*p* = 0.0467, paired t test) weeks old, respectively. (Graphs depicted with mean ± SEM).

We found that glucose tolerance was improved due to ampicillin treatment in 5 and 11 weeks old animals (*p* = 0.0114 and *p* = 0.0095, respectively, Fig 3A–3B and 3D–3E) whereas no difference in glucose tolerance was observed in the 16 weeks old animals (Fig 3C–3F). When assessing data over time (Fig 3G–3J) a significantly better glucose tolerance was observed in the young ampicillin-treated animals 5 weeks of age, as compared with data on the same animals at older ages (11 and 16 weeks of age; *p* = 0.0210 and *p* = 0.0467, respectively, Fig 3J).

The gluten-free diet did not alter glucose tolerance in the animals, but at 11 and 16 weeks of age, a significant interaction between gliadin and ampicillin was observed (*p* = 0.0099 and *p* = 0 0.0338 respectively), suggesting that gliadin at these time points might have a differential impact on glucose tolerance depending on the presence or absence of ampicillin

**Fig 4:**
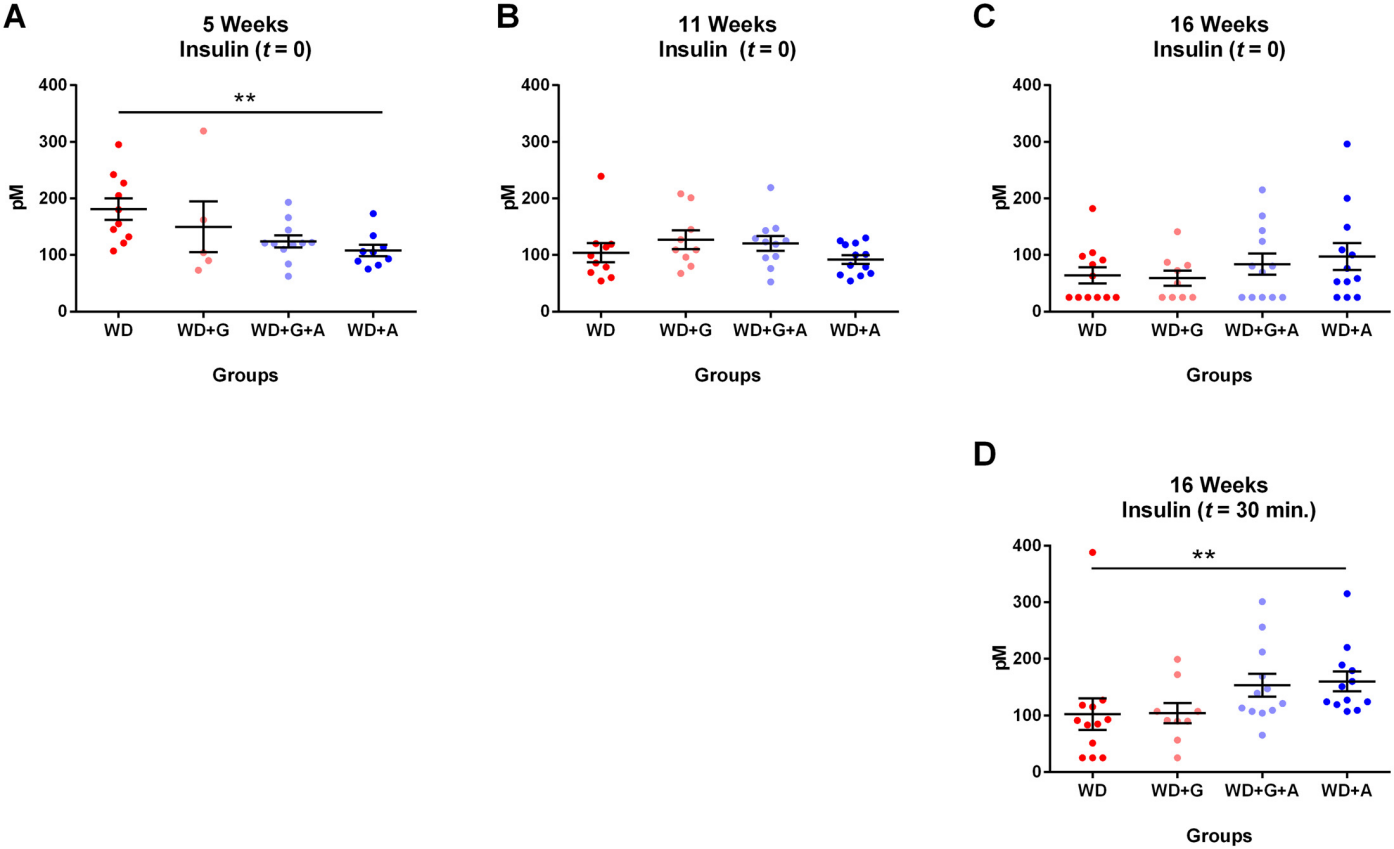
Insulin levels are lowered by ampicillin, but not by a gluten-free diet. **A-C** Insulin levels (t = 0) differed significantly between the groups at 5weeks of age with ampicillin-treated animals having lower levels than untreated animals (*p* = 0.0221, Kruskal-Wallis test (WD vs. WD+A)), whereas no differences could be demonstrated between the groups at 11 or 16 weeks of age. **D** At 16 weeks of age ampicillin-treated animals had significantly increased insulin levels 30 minutes after oral glucose administration compared to untreated animals (*p* = 0.0051, Kruskal-Wallis test (WD vs. WD+A)). Gliadin did not influence insulin levels at any time point. (Graphs depicted with mean ± SEM).

Insulin data mirrored glucose data closely, as ampicillin-treated animals had significantly lower basal insulin levels early in life than non-treated animals (WD vs. WD+A at five weeks of age; *p* = 0.0221, Fig 4A), an effect which was lost as the animals grew older (Fig 4B and 4C). Due to the small body size of the animals, repeated insulin measurements during OGTTs were only possible in the 16 weeks old animals in this study. We found a significant increase in insulin levels 30 minutes after glucose administration in the ampicillin-treated animals compared to the untreated animals (*p* = 0.0051, Fig 4D). Gluten-free diet had no effect on insulin levels, as compared to the gliadin-supplemented diets. No differences in levels of glycated hemoglobin (HbA1c) could be demonstrated between the groups and all measurements fell within the normal physiological range (S1 Fig). Furthermore, there were no differences between the four groups in plasma endotoxin or cytokine levels (S2 and S3 Figs). Together, these results suggested that altered GM composition induced by ampicillin-treatment had beneficial effects on glucose metabolism, without reducing systemic inflammation.

### Ampicillin-treated *Apoe*^*-/-*^ mice had lower cholesterol, LDL and VLDL cholesterol levels

**Fig 5:**
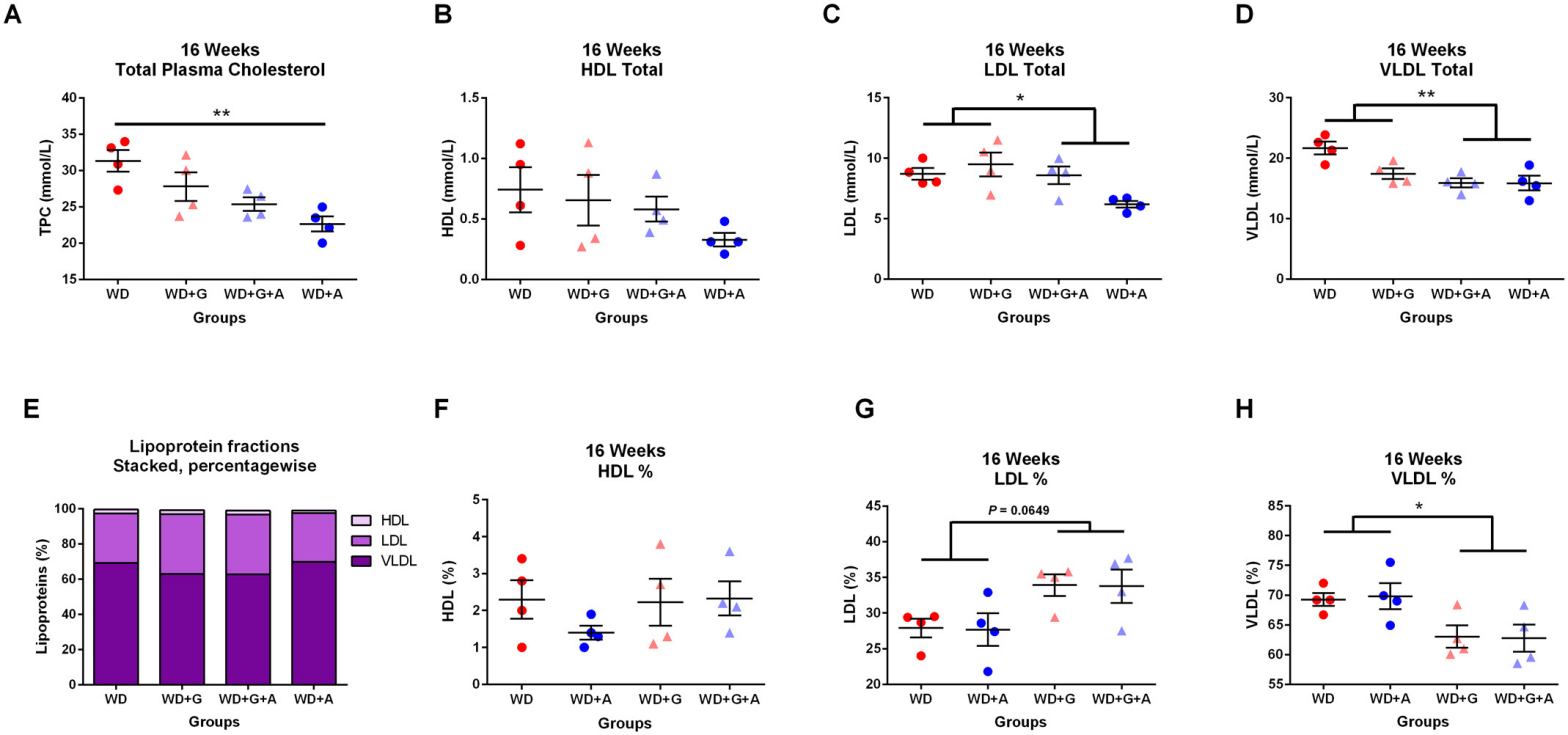
Total plasma cholesterol, LDL and VLDL are reduced by ampicillin, but not by a gluten-free diet. **A** Total plasma cholesterol levels measured at 16 weeks of age showed significantly lower levels in ampicillin-treated animals compared to untreated animals (WD vs. WD+A; *p* = 0.0064). **B-D** Lipoproteins (total values) showing significantly group differences in LDL and VLDL levels (*p* = 0.0320 and *p* = 0.0096 respectively).**E** Percentage-wise distribution (stacked) of the lipoprotein fractions HDL, LDL and VLDL. **F-H** Lipoprotein fractions (percentages) showing significantly lower VLDL percentages in animals receiving gliadin in their diet compared to gluten-free fed animals (*p* = 0.0161). Dots represent pooling of samples from two animals. Due to small group sizes (n = 8 in all four groups) data were analysed using Kruskal-Wallis test with Dunn’s multiple comparisons test with WD as control group. (Graphs depicted with mean ± SEM).

TPC measured in the 16 weeks old animals showed significantly lower cholesterol levels in ampicillin-treated animals, as compared with untreated animals (*p* = 0.0064, Fig 5A), which was caused by significantly different levels of LDL and VLDL (*p* = 0.0320 and *p* = 0.0096 respectively, Fig 5C and 5D). No difference in HDL levels could be demonstrated (Fig 5B). The absence of gluten had no significant effect on plasma lipid levels, as compared to the gliadin-supplemented diet (Fig 5A–5E). These results demonstrated that ampicillin-treatment had beneficial effects on LDL and VLDL levels.

### Gliadin shifted the ratio between LDL and VLDL in *ApoE*^*-/-*^ mice

Dietary gliadin had a significant effect on VLDL fractions (%) in plasma (Fig 5E–5H). No difference could be shown in HDL fractions between the groups, but gliadin tended to increase the % of LDL cholesterol (*p* = 0.0649, Fig 5G) and resulted in a significant reduction of the % of VLDL cholesterol (*p* = 0.0161, Fig 5H).

### Ampicillin-treatment of *Apoe*^*-/-*^ mice resulted in protection from aortic lesion development, whereas a gluten-free diet had no beneficial effect

**Fig 6:**
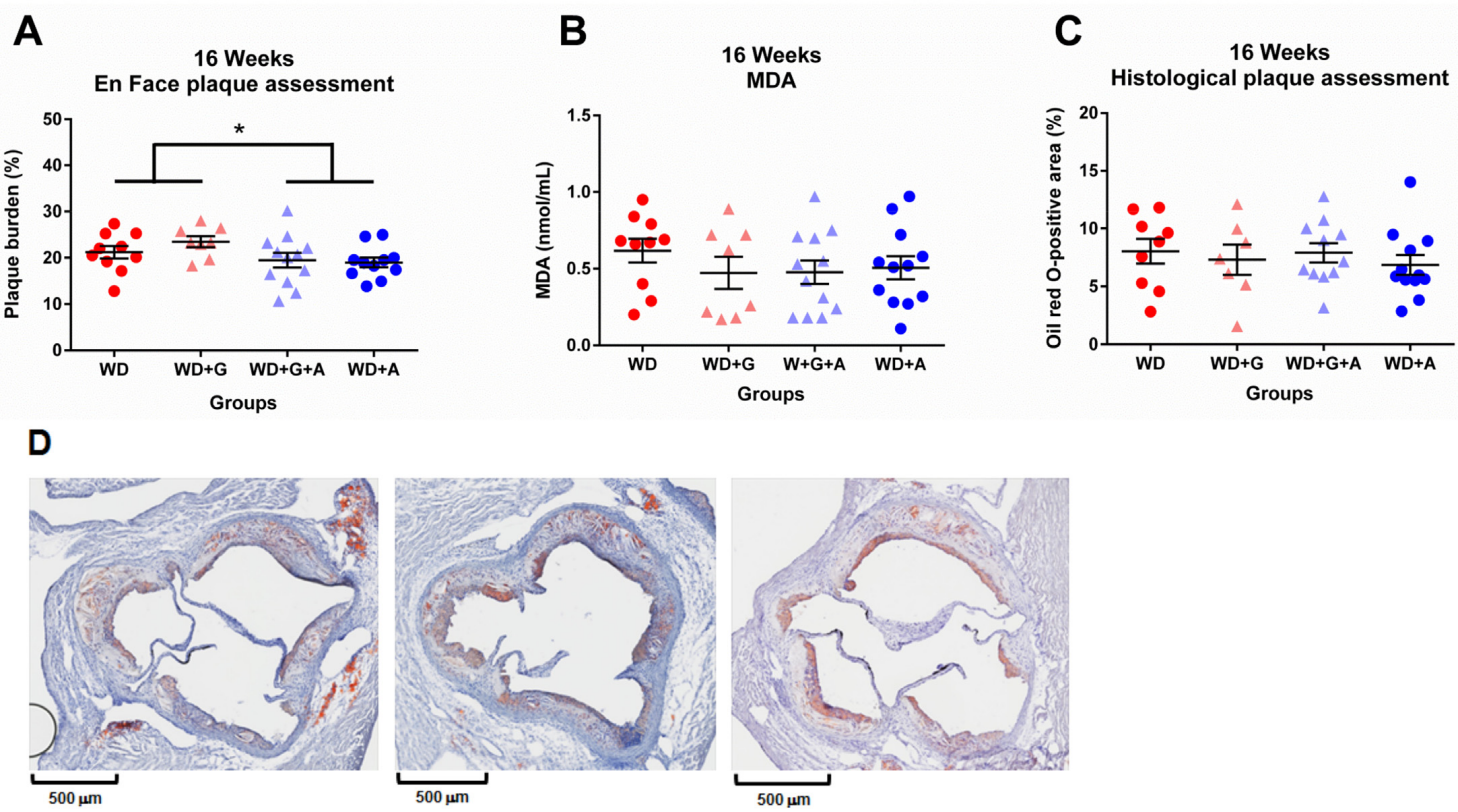
Ampicillin reduces aortic atherosclerosis without affecting MDA levels. **A** Plaque burden assessed with the “en face”-method showed significantly lower plaque burden in the thoracic aorta in ampicillin-treated animals compared to untreated animals (*p* = 0.0285). **B** MDA levels measured at 16 weeks of age did not show any differences between the groups. **C** Histological assessment of the oil red O-positive area at the aortic sinus did not show any differences between the groups. **D** Histological slide of three representative aortic sinus lesions, demonstrating complex lesions with cholesterol clefts and necrotic cores (40X, Stained with Oil Red O). (Graphs depicted with mean ± SEM).

The results discussed above show that ampicillin-treatment improved several risk factors of cardiovascular disease. In order to evaluate whether ampicillin or the gluten-free diet, as compared with the gliadin-supplemented diet, would affect atherosclerosis in *Apoe*^*-/-*^ mice, we measured atherosclerotic lesion area in *en face* thoracic aorta preparations and neutral lipid accumulation in the aortic sinus. Consistent with the reduced levels of LDL and VLDL, ampicillin-treated mice exhibited smaller atherosclerotic lesions in the thoracic aorta (*p* = 0.0285, Fig 6A). Due to the fact that plasma samples were pooled from several animals for the lipoprotein analysis, it was not possible to correlate the levels of LDL and VLDL with atherosclerosis. The gluten-free diet had no beneficial effects on atherosclerosis, as compared with the gliadin-supplemented diet (Fig 6A).

The ability of ampicillin to exert protective effects on atherosclerosis did not appear to be due to improved oxidative stress, measured as MDA levels (Fig 6B), or reduced neutral lipid accumulation in lesions, measured as oil red O-staining of lesions in the aortic sinus (Fig 6C). Representative cross sections of sinus lesions are shown in Fig 6D. These lesions exhibited features of complex lesions with necrotic cores and cholesterol clefts. Furthermore, levels of mRNA of several cytokines and other mediators of atherosclerosis were largely unchanged in mice treated with ampicillin, except for reductions in aortic *Ccl2* and *Icam1* mRNA levels (S4 Fig). No differences in inflammatory markers or LPS could be demonstrated in this study (S2, S3 and S4 Figs). Thus, ampicillin-treatment was likely to reduce atherosclerosis at least in part by lowering LDL and VLDL levels and perhaps by reducing inflammatory mediators in the artery wall.

## Discussion

The findings of this study demonstrated that a gluten-free diet, as compared to a diet supplemented with one of the two major peptides present in gluten, gliadin, had transient effects on GM composition, but did not significantly alter the cardiovascular risk factors we measured or atherosclerosis in *Apoe*^*-/-*^ mice. The effects of gliadin on GM composition are supported by previous studies [27]. Although we have previously shown a more prolonged effect of gluten on GM composition by culturing GM *in vitro* [28], in this study, gliadin lost its impact at 16 weeks of age. Natural changes in GM occurring with age may influence the metabolic measurements such as fasting blood glucose values. Gluten-free diets have received extensive interest by the popular press, a multibillion-dollar food industry, and an increasing proportion of individuals without coelic disease as the latest trend in healthy diets. Whereas it is clear that patients with coeliac disease and gluten intolerance benefit from such a diet, the beneficial effects in individuals without coeliac disease on cardiovascular risk is unknown. Our study failed to identify any positive effects of a gluten-free diet in *Apoe*^*-/-*^ mice. One potential caveat is that we only tested one of the two major peptides present in gluten. Although gliadins are believed to be the most likely gluten component to exert inflammatory effects, we cannot rule out that other components of gluten, e.g. glutinins would have an atherogenic effect. Furthermore, our study was performed using the *Apoe*^-/-^ mouse, one of the most used animal models for investigating pathophysiological as well as mechanistic aspects of atherosclerosis development since its creation in the mid-nineties [29,30]. As with all animal models developed to mimic human disease, the *Apoe*^-/-^ mouse pose a number of translational issues, and compared to humans the *Apoe*^-/-^ mouse differs in cholesterol metabolism, lipid profile and plaque pathology including lack of progression into thrombotic occlusions [29]. Our findings in this mouse model would therefore have to be validated in human subjects.

In contrast to our findings on gluten-free diets and gliadin-supplemented diets, ampicillin caused marked and sustained changes in GM composition, consistent with previous studies [28,31–34]. Ampicillin is a broad spectrum antibiotic eradicating primarily Gram positive but also Gram negative bacteria and its altering effects on GM composition is therefore not surprising [33]. GM is capable of modulating the dietary lipid digestion and uptake both directly and indirectly via metabolites [35]. For instance, GM-derived exopolysaccharides have clearly documented hypocholesterolemic effects [36]. In a study by Wang et al the important role of GM in formation of the atherogenic metabolite trimethylamine *N*-oxide from dietary choline was demonstrated [5]. Our findings of reduced aortic atherosclerosis in ampicillin-treated mice are in agreement with these previous studies. Antibiotic treatment has previously been shown to have growth enhancing abilities [31,37], which was also confirmed by our study. The effects of ampicillin on plasma lipid levels is more complex. Ampicillin has been reported to result in cholesterol accumulation, and in a study by Miyata and colleagues, using smaller group sizes of chow fed male farnesoid X receptor (Fxr)-null mice and wild-type C57BL/6N mice, ampicillin treatment resulted in increased plasma cholesterol concomitant with reduced fecal cholesterol excretion [38]. These reports tend to contradict our findings of an apparent cholesterol lowering effect of ampicillin, but the role of GM in lipid metabolism is complex and the reason for the apparent beneficial effect on metabolic parameters in this study may have been a result of the altered GM composition that we also observed. It has been suggested by others that the GM, through initiation of systemic inflammation, contribute to development of atherosclerosis and that antibiotic treatment reduces LPS levels as well as inflammation in mesenteric adipose tissue [11,34]. However, no differences, or minimal differences, in inflammatory markers, LPS or the oxidative stress marker MDA could be demonstrated in our study, and therefore, the reduction in plasma cholesterol levels may be a more reasonable explanation for our findings. Precisely how GM-alterations mediated the effects on glucose tolerance, plasma cholesterol levels and atherosclerosis needs further investigation.

In line with previous findings [32–34,39,40], ampicillin improved glucose tolerance in the early life of the animals. The lower basal insulin levels of ampicillin treated animals were lost over time. This is in accordance with one of our previous studies, in which ampicillin, if given for a shorter period after birth or at twelve weeks of age, only had a reducing impact on glucose tolerance in the early period [32]. Therefore, the effect observed later in life in studies with longer duration of treatment regimens [39,41] probably was due to the treatment being initiated in early life. Depletion of the Gram negative component of the GM has been shown to result in improved glucose tolerance in mice [39,41], and as proposed in a previous study [32], this improvement may simply be due to a decreased number of Gram-negative bacteria leading to reduced transfer of LPS to the systemic circulation, although we were not able to demonstrate differences in LPS levels in plasma.

## Conclusions

In this study we showed that a gluten-free diet had no beneficial effects on atherosclerosis or several CVD risk factors in the *Apoe*^*-/-*^ mouse model of atherosclerosis, but that sustained alteration of GM composition with a broad-spectrum antibiotic had beneficial effects on CVD risk factors as well as atherosclerosis. These findings support the concept that altering the microbiota might provide novel treatment strategies for CVD and suggest that GM composition should not be ignored or underestimated in studies on metabolic disease or atherosclerosis.

## Supporting Information

S1 Fig Glycated hemoglobin (HbA1c). HbA1c was measured three times during the study; when the animals were 5, 11 and 16 weeks of age. Whole-blood samples were collected from the tail vein and stabilized in Hemolyzing Reagent (Roche/Hitachi, Mannheim, Germany) before being analysed on a Cobas 6000 according to manufacturer’s instructions. No differences could be demonstrated between the groups and all measurements fell within the normal physiological range.
(TIF)

S2 Fig Lipopolysaccharide (LPS). LPS was measured three times during the study; when the animals were five, 11 and 16 weeks of age. Serum samples were analysed using the Plasma contents of LPS were measured using the Pyro-Gene Recombinant Factor C Endotoxin Detection System (Lonza, Basel, Switzerland) according to manufacturer’s instructions and as previously described [32]. No differences could be demonstrated between the groups.
(TIF)

S3 Fig Plasma cytokines. Plasma cytokine levels (IL-1α, IL-2, IL-4, IL-5, IL-6, IL-10, IL-12p70, IL-17, IL-18, GM-CSF, INFγ, and TNFα) were measured three times during the study; when the animals were five, 11 and 16 weeks of age. Plasma cytokines were measured with the Mouse Th1/Th2 10plex Kit FlowCytomix (#BMS820FF), Mouse IL-12p70 FlowCytomix Simplex Kit (#BMS86004FF) and Mouse IL-18 FlowCytomix Simplex Kit (#BMS8618FF, all eBioscience, Bender MedSystems GmbH, Vienna, Austria). The assay was performed according to manufacturer’s instructions. Analysis was run as previously described [32] on a BD FacsCanto Flow Cytometer (BD Biosciences, Albertslund, Denmark) and processing of data was performed using the FlowCytomixTM Pro 2.3 Software (Bender MedSystems). Due to low sensitivity of the commercial kit used, the majority of data fell below detectable limit. With the inclusion criteria of minimum half of the samples being above detection limit within every group at a given time, it was only possible to analyse IL-5 at age 11 and 16 weeks, IL-6 at age 16 weeks and IL-10 at age 16 weeks. Due to lack of Gaussian distribution data were analysed using the non-parametric Kruskal-Wallis test with Dunn’s multiple comparisons test with WD and the control group. No significant group differences could be demonstrated.
(TIF)

S4 Fig Gene expressions in the aortic arch. Thoracic aortas were placed in RNA*later*^®^ (#R0901, Sigma, St. Louis, MO 63103, USA) and kept at -80°C. They were then analysed by Real-Time Quantitative PCR as previously described by Kanter et al [42]. 18S was used as positive control. Following genes were analysed: *Abca1*, *Abcg1*, *Acsl1*, *Ccl2*, *Cd11b*, *Cd68*, *Emr1*, *Il1b*, *Il6*, *Icam1*, *Vcam1* and *Tnfa* (S4A–S4M Fig). Primer sequences can be obtained on request. Amongst the analysed genes a significant interaction was seen in regulation of the *Abca1* gene meaning that gliadin had a different effect depending on the presence of ampicillin or vice versa (*p* = 0.0256). *Ccl2* was significantly down-regulated in the ampicillin-treated animals (WD+A) compared to the untreated animals (WD) (*p* = 0.0455). *Icam1* was significantly down-regulated in animals receiving both gliadin and ampicillin compared to the untreated animals (WD) (*p* = 0.0141). Only *Abcg1* data qualified as being normally distributed and was analysed by 2-way ANOVA. All other data were analysed by Kruskal-Wallis test with Dunn’s multiple comparison test (WD as control group).
(TIF)

## Abbreviations

ANOVA: Analysis of variance
Apo E: Apolipoprotein E
ApoE^-/-^: B6.129P2-*ApoE*^*tm1Unc*^ N11
AUC: Area under the curve
DGGE: Denaturing gradient gel electrophoresis
ELISA: Enzyme-linked immunosorbent assay
FFA: Free fatty acids
GM: Gut microbiota
HDL: High density lipoprotein
HPLC: High-performance liquid chromatography
LDL: Low density lipoprotein
LLOQ: Lower limit of quantification
LPS: Lipopolysaccharide
MDA: Malondialdehyde
MetS: Metabolic syndrome
NOD mice: Non-obese diabetic mice
OGTT: Oral glucose tolerance test
PBS: Phosphate buffered saline
PCA: Principal component analysis
PCR: Polymerase chain reaction
ROS: Reactive oxygen species
T2D: Type 2 diabetes
TLR: Toll-like receptor
TPC: Total plasma cholesterol
UPGMA: Unweighted Pair Group Method with Arithmetic averages clustering algorithm
VLDL: Very low density lipoprotein
WD: Western Diet
WD+A: Western Diet and Ampicillin treatment
WD+G: Western Diet with added gliadin
WD+G+A: Western Diet with added gliadin and Ampicillin treatment
